# Sialidase Activity in the Cervicovaginal Fluid Is Associated With Changes in Bacterial Components of *Lactobacillus*-Deprived Microbiota

**DOI:** 10.3389/fcimb.2021.813520

**Published:** 2022-01-13

**Authors:** Carolina Sanitá Tafner Ferreira, Camila Marconi, Cristina M. G. L. Parada, Jacques Ravel, Marcia Guimaraes da Silva

**Affiliations:** ^1^ Department of Pathology, Botucatu Medical School, Sao Paulo State University (UNESP), Botucatu, Brazil; ^2^ Department of Basic Pathology, Sector of Biological Sciences, Federal University of Paraná (UFPR), Curitiba, Brazil; ^3^ Department of Nursing, Botucatu Medical School, Sao Paulo State University (UNESP), Botucatu, Brazil; ^4^ Institute of Genomic Science, University of Maryland School of Medicine, Baltimore, MD, United States

**Keywords:** vaginal microbiota, sialidases, bacterial vaginosis, Gardnerella, 16S rRNA

## Abstract

**Introduction:**

Sialidase activity in the cervicovaginal fluid (CVF) is associated with microscopic findings of bacterial vaginosis (BV). Sequencing of bacterial 16S rRNA gene in vaginal samples has revealed that the majority of microscopic BV cases fit into vaginal community-state type IV (CST IV), which was recently named “molecular-BV.” Bacterial vaginosis-associated bacterial species, such as *Gardnerella* spp., may act as sources of CVF sialidases. These hydrolases lead to impairment of local immunity and enable bacterial adhesion to epithelial and biofilm formation. However, the impact of CVL sialidase on microbiota components and diversity remains unknown.

**Objective:**

To assess if CVF sialidase activity is associated with changes in bacterial components of CST IV.

**Methods:**

One hundred forty women were cross-sectionally enrolled. The presence of molecular-BV (CST IV) was assessed by V3–V4 16S rRNA sequencing (Illumina). Fluorometric assays were performed using 2-(4-methylumbelliferyl)-α-D-N-acetylneuraminic acid (MUAN) for measuring sialidase activity in CVF samples. Linear discriminant analysis effect size (LEfSe) was performed to identify the differently enriched bacterial taxa in molecular-BV according to the status of CVF sialidase activity.

**Results:**

Forty-four participants (31.4%) had molecular-BV, of which 30 (68.2%) had sialidase activity at detectable levels. A total of 24 bacterial taxa were enriched in the presence of sialidase activity, while just two taxa were enriched in sialidase-negative samples.

**Conclusion:**

Sialidase activity in molecular-BV is associated with changes in bacterial components of the local microbiome. This association should be further investigated, since it may result in diminished local defenses against pathogens.

## Introduction

Bacterial vaginosis (BV) has been acknowledged as the most prevalent dysbiotic condition of the vaginal microbiota, affecting nearly one-third of women of reproductive age (Spiegel, 1991; Marconi et al., 2015). Microscopic BV is often diagnosed using Nugent criteria and is characterized by replacement of *Lactobacillus* spp. dominance by a wide array of other bacterial types (Nugent et al., 1991; Spiegel, 1991). The presence of BV was already associated with poor pregnancy outcomes and with increased risk for several sexually transmitted infections (STI), including the human immunodeficiency virus (HIV) (Leitich and Kiss, 2007; Gallo et al., 2012; Mitchell et al., 2013). The exact components of the vaginal microbiota were described utilizing sequencing hypervariable regions of the bacterial 16s rRNA gene (Ravel et al., 2011). Studies based on this molecular approach have shown that the vaginal microbiota of virtually all reproductive-aged women fits into five bacterial community state types (CSTs), of which four (CST I, CST II, CST III, and CST V) present dominance of certain *Lactobacillus* spp. CST IV is *Lactobacillus* spp.-deprived and comprises most of the cases of BV detected by microscopy; thus, it has been named as “molecular-BV” (Ravel et al., 2011; McKinnon et al., 2019; Marconi et al., 2020).

Sialidase activity is often detected in the cervicovaginal fluid (CVF) of women with microscopic BV (Marconi et al., 2013; Santos-Greatti MM de et al., 2016). These hydrolases have a negative effect on cervicovaginal immunity since they degrade local immunoglobulin A (IgA) and vaginal mucins, likely contributing to the diminished viscosity of local secretion which results in increased vulnerability to pathogens (Lewis et al., 2012; Schwebke et al., 2014; Muzny and Schwebke, 2016). The sialidase CVF concentration is correlated with local levels of IL-1beta which may lead to tissue damage increasing the vulnerability to STIs (Marconi et al., 2013; Mitchell et al., 2013). Additionally, sialidases cleave sialic acid from the terminal glycans of glycoproteins present in the vaginal mucosa, allowing bacterial adhesion to the epithelial cells (Briselden et al., 1992). Several BV-associated species may produce sialidases. However, *Gardnerella* spp. are considered the main source of this enzyme in the cervicovaginal environment (Hardy et al., 2017; Kurukulasuriya et al., 2020). In fact, *Gardnerella* spp. have been proposed as the scaffold on vaginal mucosa for the attachment of other bacterial species, such as *Prevotella* and *Atopobium*, leading to biofilm formation (Hardy et al., 2015; Muzny and Schwebke, 2016).. Thus, by cleaving the sialic acid of epithelial cells, sialidases may facilitate the adhesion of *Gardnerella* spp. to the underlying glycan-binding sites enabling biofilm formation (Varki and Gagneux, 2012; Castro et al., 2019). Vaginal biofilms have been considered the hallmark of BV and are particularly troublesome as they hinder antibiotic action leading to persistence of BV-associated bacteria after treatment (Swidsinski et al., 2008).

Thus, considering the deleterious effects of sialidases for cervicovaginal immune defense, particularly related to local biofilm formation, as well as the intricate relation between hosts’ defenses and components of their microbiota, the aim of this study was to investigate if presence of sialidase activity in CVF is associated with differences in the bacterial composition of molecular-BV.

## Materials and Methods

### Ethics Statement and Study Population

The Ethics Review Board of the Botucatu Medical School (São Paulo State University) approved this study and consent procedures (approval number 3.095.119) and by the National Commission of Ethics in Research (Comissão Nacional de Ética em Pesquisa, approval number 294.202). The aims and procedures of the study were explained to all participants, after which each of them signed a consent term for the participation in the study. This cross-sectional study included 140 non-pregnant reproductive-aged women attending a primary healthcare clinic in Botucatu, São Paulo, Brazil, for a comprehensive study on the composition of the vaginal microbiome of Brazilian women (Marconi et al., 2020).

Women were only considered for enrollment if they were 18 years old or older, were menstruating monthly, and had their last menstrual period of at least for 5 days. Approached women were asked if they had HIV infection, intrauterine device (IUD), urinary loss, therapy with antibiotics in the prior 30 days, and sexual intercourse/vaginal douching in the prior 48 h. In case of any positive response, they were not considered for study enrollment. Women who fulfilled the inclusion criteria were interviewed face to face by a member of the research team that used a structured form that included questions for assessment of sociodemographic, behavioral, and clinical characteristics.

### Sampling Procedures

During the physical exam, nurses previously trained for this study procedures assessed vaginal pH by the direct contact of commercial pH strips (range 4.0–7.0, Merck, Darmstadt, Germany) with the vaginal wall. The mid-third portion of the vaginal wall was sampled using two swabs. The first vaginal swab was kept into Amies liquid medium (Copan, Brescia, Italy) at -80°C for molecular analysis. Another vaginal swab was smeared onto glass slides for microscopic interpretation of the vaginal microbiota using the Nugent scoring system (Nugent et al., 1991). Samples of CVF were obtained by washing the vaginal wall and posterior fornix with 3 ml of sterile NaCl 9.5% [w/v] solution, as previously standardized for measurement of sialidase activity (Marconi et al., 2013).

### Molecular Analysis of Vaginal Microbiota

Frozen vaginal samples inoculated in transport medium were thawed on ice and shaken vigorously, and the swabs were then discarded. DNA extraction was performed using the PowerSoil DNA Isolation Kit (MO BIO Laboratories, Carlsbad, CA), according to the manufacturer’s protocol. Molecular analysis was performed at the Institute for Genomic Sciences of University of Maryland (Baltimore, MD), according to Fadrosh et al. (2014) and a previous study (Marconi et al., 2020). All samples were submitted to amplification of the V3–V4 hypervariable region of the 16S rRNA gene using dual-indexed 319F and 806R sets of primers. Amplicon libraries were sequenced using the 300 PE protocol on a MiSeq equipment (Illumina, San Diego, CA). Sequences were demultiplexed and quality trimmed in QIIME (version 1.8.0), as previously described (Ramírez-Guzmán et al., 2010; Marconi et al., 2020). Taxonomic assignments were performed using an in-house fifth-order Markov chain model and a pre-compiled database containing all bacterial species previously observed in vaginal microbiota (Ravel et al., 2011; Gajer et al., 2012). Samples were clustered into CSTs I to V using taxonomic information, taxa abundance, and the Jensen–Shannon divergence metrics (Marconi et al., 2020).

### Measurement of Sialidase Activity

Measurement of CVF sialidase levels was performed by the conversion of the fluorogenic substrate 2-(4-methylumbelliferyl)-α-D-N-acetylneuraminic acid (MUAN; Sigma-Aldrich, St. Louis, MO), according to methods previously optimized for CVF samples (Marconi et al., 2013). Aliquots of 50 μl of CVF supernatants were transferred to a 96-well plate (OptiPlate-96F, PerkinElmer, Waltham, MA). A volume of 50 μl of 0.35% MUAN (wt/vol) in 3 mM sodium acetate (pH 4.5) was added to the samples and kept at 37°C for 30 min. A standard curve was constructed with 10 dilution points ranging from 1000.0 to 0.1 ng/ml of purified *Clostridium perfringens* neuraminidase (Sigma-Aldrich, St. Louis, MO). Fluorescence signals were detected at 450 nm emission and 365 nm excitation and filtered at the 420-nm cutoff (Epoch instrument, Biotek, Winooski, VT). Samples were considered as positive for sialidase activity when the fluorescence was above the detection limit of the assay (set at 0.1 ng/ml corresponding to the lowest point of the standard curve).

### Data Analyses

Sialidase levels in CVF were compared across the CSTs using the non-parametric Kruskal–Wallis test in Stata software (StataCorp, College Station, TX) with p-value <0.05 considered as statistically significant. The Shannon–Weiner index was calculated for assessing alpha-diversity based on median rarefied taxa reads using the *vegan* package in R (Oksanen et al., 2012) and compared between sialidase-positive and -negative samples using the Mann–Whitney test (p < 0.05 considered as significant). Relative abundances of bacterial taxa identified in molecular-BV (i.e., CST IV) were compared using linear discriminant analysis effect size (LEfSe) according to the status of CVF sialidase activity (Segata et al., 2011). Only relative abundances of microbial taxa with >0.05% population-wide representativeness were included at LEfSe.

## Results

The characteristics of the 140 participants of the study are detailed in **Supplementary Table 1**. The median age of participants was 33 years (range: 18–51). Most participants were living in a steady relationship upon study enrollment (63.6%, n = 89). A small fraction of participants reported having more than one sex partner in the prior 12 months (10%, n = 14) or having a new sex partner within the prior 4 months (16.4%, n = 23). Consistent use of condoms during the intercourse was reported by only 15.7% (n = 22) of the participants. Current use of hormonal contraceptives was reported by 42.9% (n = 60) participants. Cervical infections by *C. trachomatis* and *N. gonorrhoeae* were detected in 8 (5.7%) and 2 (7.1%) participants. As also detailed in **Supplementary Table 1**, a microscopic assessment of vaginal microbiota using the Nugent scoring criteria showed that 48 (34.3%) participants had microscopic BV.

As displayed in **Table 1**, sialidase activity was detected in 34 (24.3%) out of 140 CVF samples. The measured values of vaginal pH of sialidase-positive participants were statistically superior (median: 4.9; range: 4.7–5.8) to those with no sialidase activity (median: 4.7; range: 4.0–5.5), (p < 0.001). When observing the categories of vaginal microbiota retrieved from microscopic Nugent analysis, sialidase activity was almost exclusive to BV (91.2%, n = 31) (**Table 1**).

**Table 1:** Covariates of vaginal microbiota by the status of sialidase activity in cervicovaginal fluid samples.

Characteristics	Sialidase activity		
	Negative (n = 106)	Positive (n = 34)	p-value
Vaginal pH; median (range)	4.7 (4.0–5.5)	4.9 (4.7–5.8)	<0.0001 *^a^*
Nugent score categories; n (%)			<0.0001 *^b^*
0–3 (normal)	75 (70.8)	0 (0.0)	
4–6 (intermediate)	14 (13.2)	3 (8.8)	
7–10 (bacterial vaginosis)	17 (16.0)	31 (91.2)	

a Mann–Whitney non-parametric test.

b Chi-squared test.

p-values <0.05 considered as statistically different.

A molecular analysis of 140 vaginal samples resulted in 1,938,832 reads with a total 116 bacterial taxa identified. A total of 96 participants were clustered into *Lactobacillus*-dominant CSTs: *L. crispatus*-dominated CST I (n = 40), *L. gasseri*-dominated CST II (n = 4), *L. iners-*dominated CST III (n = 51), or *L. jensenii-*dominated CST V (n = 1). A total of 44 (31.4%) participants had molecular-BV (*Lactobacillus*-depleted CST IV). Molecular-BV was diagnosed in 41 (85.4%) out of the 48 microscopic-BV cases detected in the study (**Supplementary Figure 1**). As displayed in **Table 2**, nearly all participants with detectable levels of CVF sialidases had molecular-BV (n = 30 out of 34) (p < 0.0001). Also, significantly increased sialidase levels (expressed in ng/mL of CVF sample) were observed in molecular-BV (median 10.3, range: 0.0–818.9) when compared to *L. iners*-CST III (median: 0.0; range 0.0–84.4) (p = 0.0001).

**Table 2:** Presence of sialidase activity in the cervicovaginal fluid, as well as measured levels of sialidases, by molecularly defined community state types (CSTs).

	CST I (n = 40)	CST III (n = 51)	Molecular-BV (CST IV) (n = 44)	CSTs II and V *^a^* (n = 5)	p-value
Sialidase activity					<0.0001 *^b^*
Negative	40 (100.0)	48 (94.1)	14 (31.8)	4 (80.0)	
Positive	0 (0.0)	3 (5.9)	30 (68.2)	1 (20.0)	
Sialidases (ng/mL), median (range)	nd	0.0 (0.0–84.9)	10.5 (0.0–818.9)	0.0 (0.0–1.8)	0.0001 *^c^*

a CST II (n = 4) and CST V (n = 1) were merged into one category and excluded from analysis, as they were underrepresented in the study population.

b Chi-squared test for comparison within CSTs I, III, and IV.

c Mann–Whitney non-parametric test for comparison between CSTs III and IV.

nd, not detected.

**Figure 1:**
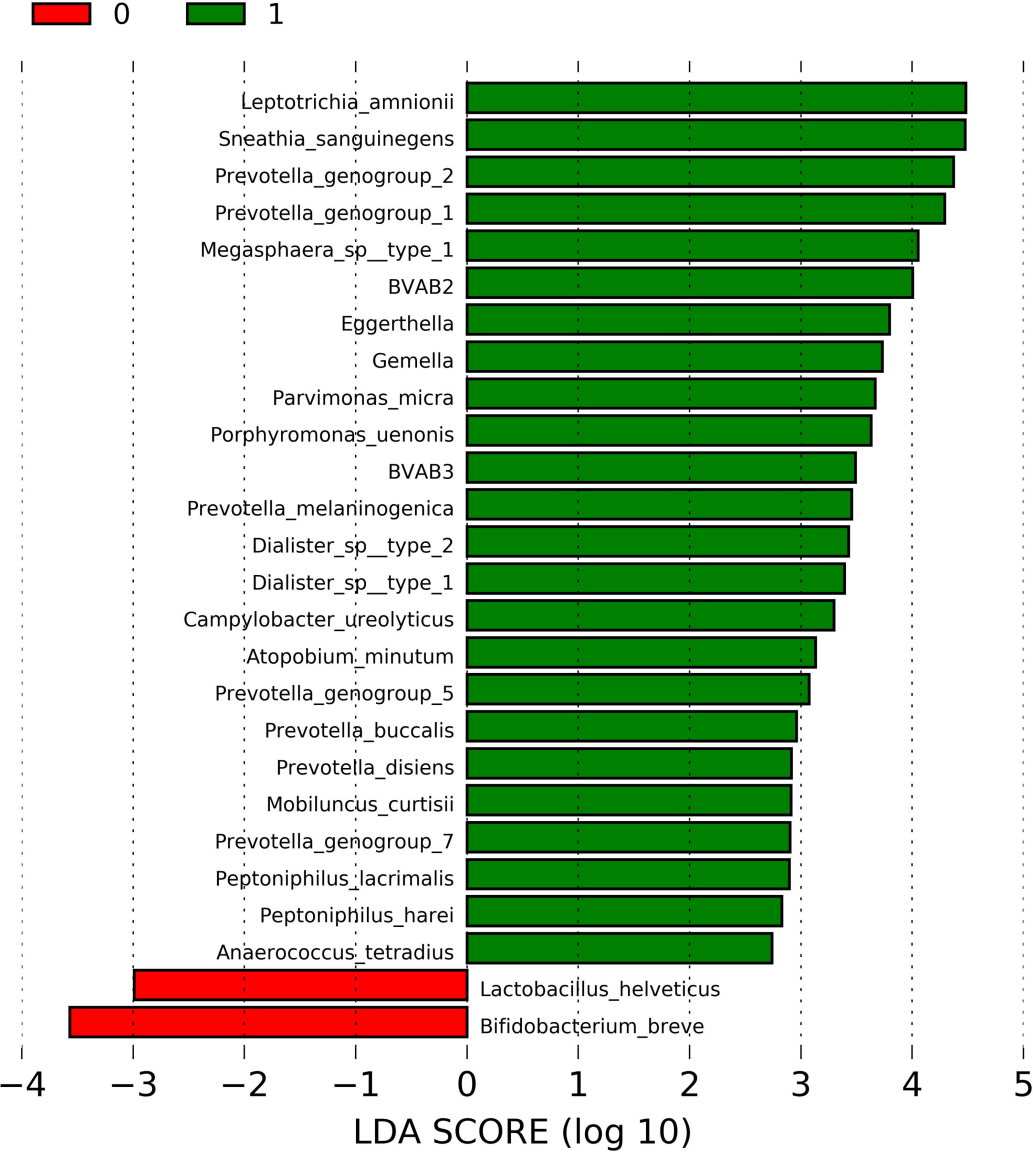
Linear discriminant analysis (LDA) effect size (LEfSe) for comparison of taxa abundance among CST IV-positive samples, according to the presence (n = 30) or absence (n = 14) of detectable levels of sialidase activity in cervicovaginal fluid. LDA scores of significantly enriched taxa in the presence of sialidase activity are represented by green bars, while red bars are referent to LDA scores of enriched taxa in sialidase-negative samples. p-values <0.05 were considered as significant.

In order to identify the bacterial taxa differently abundant in the presence of sialidase activity, the 44 samples with molecular-BV (CST IV) were tested using LEfSe. The relative abundances of 80 bacterial taxa that were the most representative population-wide (>0.05%) were used at this analysis. The list of all bacterial taxa and their respective overall abundances is provided in **Supplementary Table 2**. **Figure 1** depicts the result of LEfSe showing a total of 24 enriched taxa in the presence of sialidase activity, of which 7 (29%) belong to *Prevotella* genera. Other well-acknowledged BV-associated bacteria taxa were also enriched in sialidase-positive samples, such as *Leptotrichia amnionii*, *Megasphaera* sp., *Mobiluncus curtisii*, and BVAB3 (the novel *Mageeibacillus indolicus*). Only two taxa were enriched in sialidase-negative samples: *L. helveticus* and *Bifidobacterium breve*. *Gardnerella* spp. was not figured out among the differently enriched taxa. A comparison of the Shannon–Weiner index showed that bacterial diversity was significantly increased in the presence of sialidase activity (median: 1.90; range: 1.27–2.77) in relation to sialidase-negative samples (median: 1.16; range: 0.51–2.21) (p = 0.0001).

## Discussion

The impairment of cervicovaginal immunity due to the presence of bacterial sialidases has been investigated by several studies, while the impact of these hydrolases on local microbiota remains poorly known (Lewis et al., 2012; Muzny and Schwebke, 2016; Hardy et al., 2017; Castro et al., 2019). Thus, the current study adds to the literature novel information about the changes in the microbial components of molecular-BV when in the presence of sialidase activity. The population enrolled in this study consisted of women of reproductive age in order to minimize the well-known impact of low estrogen levels on the vaginal microbiota (Hillier and Lau, 1997).

The current results showing the association between microscopic BV and presence of sialidase activity reinforce similar reports in the literature (Briselden et al., 1992; Smayevsky et al., 2001). In fact, several BV-associated bacteria, such as *Gardnerella* spp., *Prevotella* spp., and *Bacteroides* spp., were already shown as capable of sialidase production, corroborating with this hypothesis (Briselden et al., 1992; Smayevsky et al., 2001). Despite the relevance of previous and current findings on the relation between bacterial sialidases and microscopic-BV, microscopy provides very limited information regarding the actual components of the microbiota (Briselden et al., 1992; Marconi et al., 2013). Thus, this study was based in 16S rRNA sequencing and CST assignments to classify the vaginal microbiota. These strategies provide more precise information on the bacterial taxa present and their association change with sialidase activity. Particularly, these results focused on the *Lactobacillus*-deprived CST IV, which has been acknowledged as molecular-BV (McKinnon et al., 2019). The bacterial composition of CSTs observed in this study population was described in detail in our prior study and is consistent with the recent nearest centroid classification named VALENCIA (France et al., 2020; Marconi et al., 2020).

Sialidase activity was almost exclusively observed in molecular-BV, as only 4 out of 34 sialidase-positive samples were molecularly classified as non-CST IV. These findings were not unexpected as CST IV “molecular-BV” comprises most of the cases of microscopic-BV detected by Nugent criteria (Ravel et al., 2011; Marconi et al., 2020). Although this study design does not allow to establish a causal and effect link between CVF sialidases and microbiota components, it does show that several (n = 24) bacterial taxa of molecular-BV are enriched in the presence of sialidase activity, thus contributing to a significantly increased alpha-diversity. Interestingly, although *Gardnerella* spp. are recognized as the main sialidase producers in the cervicovaginal environment (Hardy et al., 2017; Kurukulasuriya et al., 2020), they were not figured out among the differently enriched taxa. Recently, it has been demonstrated that *Gardnerella* sialidase-encoding genes nanH2 and nanH3 are more restricted to some strains than previously thought (Kurukulasuriya et al., 2020). Thus, despite the fact that sialidases contribute for *Gardnerella* adherence and biofilm formation, these species are also highly abundant in sialidase-negative CFV samples (Hardy et al., 2017).

There are several plausible hypotheses for the increased relative abundance of certain taxa in the presence of CVF sialidase activity. Firstly, non-*Gardnerella* species might also be acting as local sources of sialidases, such as *Prevotella*. In fact, the current results show that *Prevotella* genogroups were the most frequent taxa among those enriched in the presence of sialidases (7 out of 24). Thus, increased sialidases may also be due to the higher abundance of *Prevotella* sialidase-producing strains. In fact, an early study by Briselden et al. (1992) showed that the majority of *Prevotella* sp. isolates from vaginal cultures are sialidase producers (Briselden et al., 1992). Interestingly, the latter study also showed that few *G. vaginalis* isolates are sialidase producers, which also corroborates with recent findings showing that few *Gardnerella* strains present the sialidase-encoding genes.

Increased bacterial diversity in the presence of sialidases may also be due to their impairment of local immunity, allowing the overgrowth of several bacterial species that overcome the host’s defenses (Lewis et al., 2012; Lewis et al., 2013; Schwebke et al., 2014). Also, the increased proinflammatory cytokine levels observed in the presence of CVF sialidase activity may lead to increased risk for HIV acquisition (Mitchell et al., 2013). Moreover, sialidases are associated with the early stages of vaginal biofilm formation, as they enable the adherence of *Gardnerella* sp. to glycan-binding sites of epithelial cells uncovered by the removal of sialic acids (Varki and Gagneux, 2012; Muzny and Schwebke, 2016; Hardy et al., 2017). Biofilm-forming *Gardnerella* sp. would then act as scaffold for attachment of other BV-associated species, such as *Atopobium vaginae* and *Prevotella* sp., among others. Current knowledge on microbial composition of vaginal biofilms points out to a predominance of *Gardnerella* sp. and *A. vaginae*. However, other BV-associated bacteria such as those enriched in sialidase activity may also be figured out as secondary components of vaginal biofilms, warranting future investigations (Hardy et al., 2015; Castro et al., 2019). A recent study by Castro et al. (2019) showed that several BV-associated bacteria increase the expression of the *Gardnerella* sp. sialidase-encoding gene in an *in vitro* dual-species biofilm model (Castro et al., 2019). However, the latter study did not test the bacterial taxa that were enriched in the presence of sialidase in the current study, the exception being for *P. bivia*. Therefore, further studies based on polymicrobial biofilm models should target other bacterial types associated with dysbiotic vaginal microbiota, especially those enriched in the presence of sialidase activity.

Regarding the only two taxa that were enriched in sialidase-negative samples, one belongs to *Lactobacillus* genera, *L. helveticus*, which is widely accepted as beneficial to vaginal microbiota. Despite the characteristic paucity of *Lactobacillus* spp. in CST IV, these organisms are frequently detected in low abundances in this community (Ravel et al., 2011; Marconi et al., 2020). Additionally, *Bifidobacterium breve* was enriched in samples with no sialidase activity. Despite being typically associated with gut microbiota, some *Bifidobacterium* spp., including *B. breve*, were already shown as frequent and abundant colonizers of the vaginal microbiota (Freitas and Hill, 2017). Also, vaginal isolates of *B. breve* produce L-lactic acid *in vitro* (Freitas and Hill, 2017), which may contribute to a low pH inhibiting the growth of sialidase-producing BV-associated bacteria.

In conclusion, this study shows that the presence of CVF sialidase activity is associated with changes in bacterial composition of molecular-BV characterized by increased bacterial diversity and abundance of several BV-associated bacteria, but not *Gardnerella* spp. Thus, these results may serve as basis for the better understanding on how virulence factors produced by major constituents of vaginal biofilms (i.e., *Gardnerella* spp.) may affect the local microbiota.

## Data Availability Statement

The datasets presented in this study can be found in online repositories. The names of the repository/repositories and accession number(s) can be found as follows: http://hdl.handle.net/11449/215315.

## Ethics Statement

This study involving human participants was reviewed and approved by the Ethics Review Board of the Botucatu Medical School (São Paulo State University) (approval number 3.095.119)and by the National Commission of Ethics in Research (Comissão Nacional de Ética em Pesquisa, approval number 294.202). The patients/participants provided their written informed consent to participate in this study.

## Author Contributions

CSTF: enrolled study participants, performed laboratory analysis, wrote the first version of the manuscript; CM: designed the study, performed statistical analysis, reviewed the first version of the manuscript, CMLP: enrolled study participants, critically reviewed the data analysis and display; JR: performed the molecular analysis, critically reviewed the manuscript; MGS: coordinated the study, critically reviewed the data analysis and display.

## Funding

Financial support was granted by São Paulo Research Foundation (FAPESP) (grants #2012/16800-3 and #2012/10403-2) to MS and CM and by the Coordenação de Aperfeiçoamento de Pessoal de Nível Superior (CAPES), Brazil – Finance code 001 by providing a doctorate scholarship to CF.

## Conflict of Interest

The authors declare that the research was conducted in the absence of any commercial or financial relationships that could be construed as a potential conflict of interest.

## Publisher’s Note

All claims expressed in this article are solely those of the authors and do not necessarily represent those of their affiliated organizations, or those of the publisher, the editors and the reviewers. Any product that may be evaluated in this article, or claim that may be made by its manufacturer, is not guaranteed or endorsed by the publisher.
