# Supplementary material for: Sialidase Activity in the Cervicovaginal Fluid Is Associated With Changes in Bacterial Components of Lactobacillus-Deprived Microbiota
Source: Front Cell Infect Microbiol. 2022 Jan 13;11:813520. doi: 10.3389/fcimb.2021.813520 (PMC8793624; doi:10.3389/fcimb.2021.813520)

Supplementary tables

Supplementary table 1. Demographic, behavioral and clinical characteristics of the 140 study participants.

Characteristics	N or median	% or range
**Age** (years); median (range)	33	18 - 51
**Ethnicity**		
White	80	57.1
Non-white	60	42.9
**Body mass index**[a]; median (range)	25.9	17.6 - 53.0
**Marital status**		
Single	51	36.4
In a steady relationship (living with partner)	89	63.6
**Years at school**[b]; median (range)	9	0-16
**Has any personal income**[c]		
No	57	40.7
Yes	83	59.3
**Smoker**		
No	110	78.6
Yes	30	21.4
**Number of sex partner** (prior 12 months)		
0 or 1	126	90.0
2 or more	14	10.0
**New sex partner** (prior 4 months)		
No	117	83.6
Yes	23	16.4
**Abnormal vaginal discharge treated in the past**		
No	77	55.0
Yes	63	45.0
**History of sexually transmitted infections**		
No	127	90.7
Yes	13	9.3
**Frequency of condom use**		
Never	99	70.7
Inconsistent	19	13.6
Consistent	22	15.7
**Hormonal contraceptive use**		
No	80	57.1
Oral	46	32.9
Injectable	14	10.0
**Cervical infection detected at enrollment**		
None	130	92.9
Positive for *Chlamydia trachomatis*	8	5.7
Positive for *Neisseria gonorrhoeae*	2	1.4

Characteristics	N or median	% or range
**Vaginal microbiota (Nugent scoring)**		
0-3 Normal (scores 0 - 3)	75	53.6
4-6 Intermediate (scores 4 - 6)	17	12.1
7-10 Bacterial vaginosis (scores 7 - 10)	48	34.3

[a] BMI: body mass index defined as weight (kg) divided by height squared (meters).
[b] Number of school grades approved.
[c] Has a personal income from a formal or informal job.

z

Supplementary Table 2. Overall relative abundances (RAs) of taxa retrieved from molecular assessment of vaginal samples. Overall RAs were obtained by the sum of median (=10784) rarefied read counts of each taxon across samples. Sum of each taxon was then divided by total read counts of the study (2139017), those with overall RAs <0.001 were not included at LEfSe (red).

**Taxa**	**Overall RA** (%)	**Cumulative overall RA** (%)
Lactobacillusiners	30.777919	
Lactobacilluscrispatus	23.707339	54.48526
Gardnerellavaginalis	8.441398	62.92666
BVAB1	5.107998	68.03465
Atopobiumvaginae	4.218023	72.25268
Lactobacillusgasseri	4.199165	76.45184
Lactobacillusjensenii	3.305398	79.75724
*Megasphaera*sp.type1	2.891484	82.64872
Sneathiasanguinegens	2.548393	85.19712
Lactobacillushelveticus	2.222530	87.41965
Leptotrichiaamnionii	1.862793	89.28244
BVAB2	1.261917	90.54436
*Prevotella*genogroup1	1.086990	91.63135
*Prevotella*genogroup2	0.944979	92.57633
Parvimonasmicra	0.634080	93.21041
*Dialister*sp.type2	0.604308	93.81471
*Dialister*sp.type1	0.604234	94.41895
Mycoplasmahominis	0.447787	94.86673
*Prevotella*genogroup3	0.403197	95.26993
Gemella	0.374366	95.64430
Raoultellaplanticola	0.362705	96.00700
Bifidobacteriumbreve	0.322009	96.32901
Porphyromonasbennonis	0.252623	96.58163
Streptococcusagalactiae	0.251228	96.83286
Fusobacteriumnucleatum	0.245746	97.07861
Mobiluncusmulieris	0.222894	97.30150
BVAB3	0.205165	97.50667

**Taxa**	**Overall RA** (%)	**Cumulative overall RA** (%)
Lactobacillusvaginalis	0.197353	97.70402
Sutterellastercoricanis	0.182583	97.88660
Prevotellamelaninogenica	0.179294	98.06590
Arcanobacteriumphocae	0.177894	98.24379
Eggerthella	0.151240	98.39503
Agrobacteriumtumefaciens	0.129612	98.52464
Finegoldiamagna	0.112550	98.63719
Clostridiumcolicanis	0.108733	98.74593
Howardellaureilytica	0.106107	98.85203
*Megasphaera*sp.type2	0.104877	98.95691
Prevotellabivia	0.103267	99.06018
Ureaplasmaparvum	0.097113	99.15729
*Prevotella*genogroup4	0.084164	99.24145
Peptoniphilusharei	0.069311	99.31077
Anaerococcustetradius	0.057485	99.36825
Arcanobacteriumhippocoleae	0.050693	99.41894
Porphyromonasuenonis	0.048448	99.46739
Prevotellabuccalis	0.043339	99.51073
Peptostreptococcusanaerobius	0.041611	99.55234
Streptococcusoralis	0.038228	99.59057
Sutterellamorbirenis	0.037930	99.62850
Lactobacilluscoleohominis	0.033233	99.66173
Atopobiumminutum	0.026683	99.68841
CandidateDivisionTM7vaginal	0.026461	99.71488
Aerococcuschristensenii	0.026210	99.74109
Peptoniphilusindolicus	0.025000	99.76609
Dialisterpropionicifaciens	0.024188	99.79027
Bacteroidesuniformis	0.023524	99.81380
Peptoniphiluslacrimalis	0.020093	99.83389
Porphyromonasendodontalis	0.019982	99.85387
Prevotelladisiens	0.016955	99.87083
Porphyromonassp.type1	0.014596	99.88542
*Prevotella*genogroup5	0.010780	99.89620
Peptostreptococcusstomatis	0.008597	99.90480
Peptoniphilusasaccharolyticus	0.008165	99.91297
Anaerococcusvaginalis	0.007989	99.92095
Pseudomonasfluorescens	0.007121	99.92808
*Prevotella*genogroup7	0.007070	99.93515
Streptococcusanginosus	0.006958	99.94210
Bifidobacteriumlongum	0.006152	99.94826
Enterococcusfaecalis	0.005244	99.95350
Veillonellaatypica	0.005010	99.95851

**Taxa**	**Overall RA** (%)	**Cumulative overall RA** (%)
Roseburiafaecis	0.004785	99.96329
Prevotellagenogroup6	0.003301	99.96660
Lactobacillusmucosae	0.003279	99.96987
Mobiluncuscurtisii	0.003067	99.97294
Streptococcussalivarius	0.003058	99.97600
Varibaculumcambriense	0.002839	99.97884
Staphylococcushominis	0.002443	99.98128
Mycoplasmagenitalium	0.002217	99.98350
Corynebacteriumaccolens	0.001770	99.98527
Staphylococcusepidermidis	0.001516	99.98678
Veillonellaparvula	0.001431	99.98822
Alloscardoviaomnicolens	0.001133	99.98935
Campylobacterureolyticus	0.001077	99.99043
Actinomycesmeyeri	0.000984	
Staphylococcuslugdunensis	0.000937	
Actinomycesurogenitalis	0.000802	
Veillonellamontpellierensis	0.000778	
Aerococcusviridans	0.000756	
Eubacteriumsaphenum	0.000687	
Streptococcusequinus	0.000662	
Shuttleworthiasatelles	0.000587	
Bifidobacteriumbifidum	0.000488	
Catonellamorbi	0.000479	
Atopobiumrimae	0.000416	
Streptococcussanguinis	0.000365	
Staphylococcushaemolyticus	0.000344	
Weissellaparamesenteroides	0.000335	
Acinetobacterbaumannii	0.000219	
Escherichiacoli	0.000175	
Eubacteriumrectale	0.000098	
Arcanobacteriumhaemolyticum	0.000095	
Sutterellawadsworthensis	0.000093	
Pseudomonasaeruginosa	0.000084	
Lactobacillusrhamnosus	0.000081	
Staphylococcuswarneri	0.000028	
Actinomycesneuii	0.000028	
Pediococcusacidilactici	0.000028	
Enterococcusfaecium	0.000015	
Lactobacilluscasei	0.000011	
Dialistersp.type3	<0.000001	
Streptococcusparasanguinis	<0.000001	
Eubacteriumeligens	<0.000001	

**Taxa**	**Overall RA** (%)	**Cumulative overall RA** (%)
Lactobacillussalivarius	<0.000001	
Staphylococcusaureus	<0.000001	
Acinetobactercalcoaceticus	<0.000001	
Pseudomonasstutzeri	<0.000001	
Eubacteriumsiraeum	<0.000001	

Supplementary Figure 1. Venns´s diagram showing the distribuition of the 140 study participants according to the diagnosis of microscopic- and molecular-bacterial vaginosis (BV).

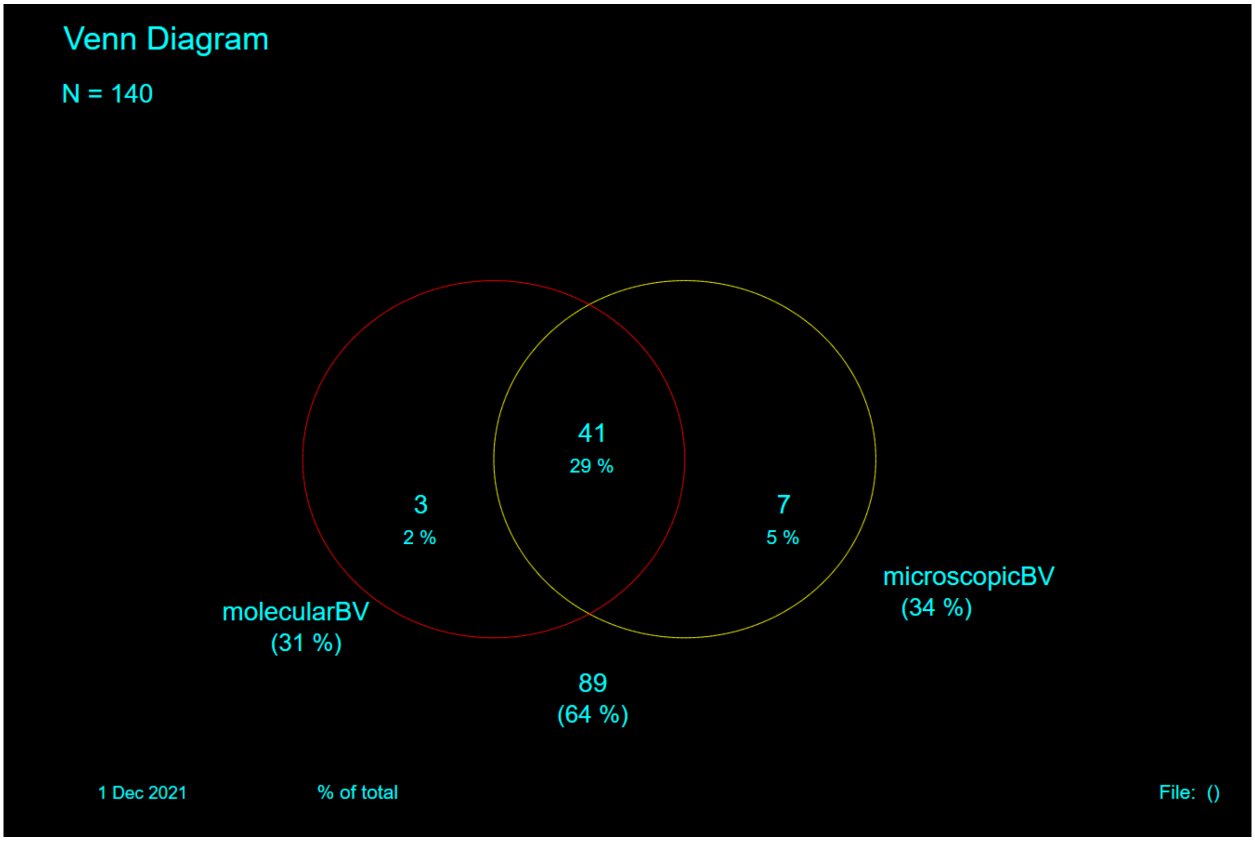

Supplement: Supplementary file 1 [file DataSheet_1.pdf]
